# Beta 2-adrenergic pathway combats Alzheimer’s disease: Restoring cognition and synaptic integrity

**DOI:** 10.4103/NRR.NRR-D-25-00529

**Published:** 2025-10-30

**Authors:** Shaomin Li, Shan-Xue Jin

**Affiliations:** Ann Romney Center for Neurologic Diseases, Brigham and Women’s Hospital and Harvard Medical School, Boston, MA, USA

**Keywords:** Alzheimer’s disease, amyloid-beta, β_2_-adrenergic receptor, DNA methylation, enriched environments, histone modification, long-term potentiation, microglia, microRNAs, mitochondria, neuroinflammation, synaptic plasticity

## Abstract

Alzheimer’s disease is typified by amyloid-beta oligomer-mediated synaptic disruption, neuroinflammation, and mitochondrial loss of function, culminating in cognitive decline. Recent evidence points toward the β_2_-adrenergic receptor as a target through its regulation of synaptic plasticity, neuroinflammation, and epigenetic control. Activation of β_2_-adrenergic receptor potentiates long-term potentiation, reverses amyloid-beta-mediated synaptic loss, and stimulates neuroprotective gene expression through cyclic adenosine monophosphate-protein kinase A-cyclic AMP response element-binding protein. Moreover, β_2_-adrenergic receptor suppression of histone deacetylase 2/3 promotes transcriptional reprogramming, supporting synaptic function. Beyond synaptic maintenance, activation of β_2_-adrenergic receptor prevents neuroinflammation by polarizing microglia toward an anti-inflammatory phenotype and augmenting amyloid-beta degradation. Additionally, mitochondrial metabolism is regulated by β_2_-adrenergic receptor, diminishing oxidative stress and allowing for bioenergetic resilience. Enriched environments mediate their neuroprotective effects through, in part, activation of β_2_-adrenergic receptor, supporting its role in promoting synaptic resilience. Pharmacological activation of β_2_-adrenergic receptor with specific agonists such as formoterol and clenbuterol has shown promise in preclinical models of Alzheimer’s disease by restoring cognitive function and synaptic integrity. In this review, the molecular mechanisms of β_2_-adrenergic receptor-mediated neuroprotection are examined, with specific emphasis on its regulation of synaptic plasticity, neuroinflammation, mitochondrial function, and epigenetic control. Due to its multi-faceted action for maintenance of neuronal health, activation of β_2_-adrenergic receptor is an appealing therapy for Alzheimer’s disease. Future research needs to target optimizing brain-penetrant β_2_-adrenergic receptor agonists and determining their long-term effects on Alzheimer’s disease pathology.

## Introduction

The most common cause of dementia, Alzheimer’s disease (AD), affects millions of people worldwide and places a large strain on patients, caregivers, and healthcare systems. Pathologically, AD is characterized by the deposition of extracellular amyloid-beta (Aβ) plaques and intracellular neurofibrillary tangles made up of hyperphosphorylated tau protein, leading to synaptic dysfunction, neuronal loss, and cognitive deterioration. Soluble Aβ oligomers as synaptic mediators of AD toxicity have emerged as a central hypothesis in recent studies (Shankar et al., 2008; Castellani et al., 2024; Huang, 2024). The propagating toxic species disrupt neuronal communication, synaptic plasticity, and engender a loss of both structural and functional connectivity needed for cognitive processes such as learning and memory. Controversial and decades into research, no disease-modifying therapies for AD remain, which is why new therapeutic targets must be discovered.

The ability of synapses to adjust their strength, by enhancement or decrease, as a result of neural activity, is essential for supporting both learning and memory processes (Caya-Bissonnette and Beique, 2024; Hagena and Manahan-Vaughan, 2024; Navakkode and Kennedy, 2024). This process, referred to as synaptic plasticity, is critical for the reshaping of neural circuits and cognition. Long-term potentiation (LTP) and long-term depression (LTD) are typically proposed as models for time-dependent modulation of synaptic efficacy, the two main phenotypic expressions of this plastic flexibility. LTP, considered by many as a cellular correlate of learning, is characterized by long-lasting synaptic facilitation, whereas LTD is characterized by selective reduction of the strengths of synapses. Both processes are tightly tuned by firing events of neurons, activity-dependent modulation of glutamate receptors, and intracellular signaling pathways (Li and Stern, 2022; Caya-Bissonnette and Beique, 2024; Zhang et al., 2024). Inhibition of synaptic plasticity by Aβ oligomers is proposed as the initiating event of AD-related pathology. It was hypothesized that oligomers in these pathways disrupt glutamatergic receptor function, disrupt calcium homeostasis, and compromise downstream cascades of signaling necessary for synaptic stability, leading to cognitive impairments (Sivanesan et al., 2013; Li and Selkoe, 2020; Caballero et al., 2022; Martinez et al., 2024).

Catecholamine β_2_-adrenergic receptor (β_2_-AR) isoform of G protein-coupled receptor is being increasingly appreciated as a master switch of synaptic plasticity and a candidate neuroprotectant in AD (Dang et al., 2014; Xu et al., 2018; Wu et al., 2020; Jin et al., 2022, 2023; Woo et al., 2022; Li, 2023). The β_2_-AR is therefore activated to different levels via a plethora of mechanisms improving synaptic function such that cyclic adenosine monophosphate (cAMP), gene regulation can be affected (Jin et al., 2022, 2025; Li, 2023) along with activation of anti-inflammatory pathways and also maintains in a previously described mechanism (Christiansen et al., 2011; Sharma et al., 2019; Jin et al., 2022; Ireton et al., 2023; Li, 2023). Synaptically, β_2_-AR activation enhances LTP and protects from Aβ-induced synaptic perturbation by modulating cAMP-dependent protein kinase A (PKA) pathways (Man et al., 2020; Jin et al., 2022; Ireton et al., 2023; Larsen et al., 2023; Li, 2023). Furthermore, β_2_-AR activation increases neuroprotective genes such as the brain-derived neurotrophic factor (BDNF) (Mellios et al., 2014; Han et al., 2019) and strengthens mitochondrial activity (Lim et al., 2021; Chai et al., 2022; Arif et al., 2024; Chang et al., 2024), which are necessary to sustain synaptic resilience under stress.

In addition to its known synaptic functions, β_2_-AR plays a role in inflammation and epigenetic programming. Activation of microglial β_2_-AR inhibits the production of pro-inflammatory cytokines, resisting neuroinflammation, the hallmark of AD (Xu et al., 2018; Evans et al., 2024; Majewska et al., 2024). Conversely, recent work has demonstrated that β_2_-AR controls epigenetic regulation through both the tetrameric stabilization of histone deacetylases (HDACs) (Yuliana et al., 2018) and microRNAs (miRNA) (Mellios et al., 2014; Mostafavi et al., 2014), which directly or indirectly participate in synaptic plasticity and memory. β_2_-AR signaling has such numerous effects on cognition, which suggest that targeting this receptor for treating the heterogeneous pathophysiology of AD may be a tempting proposition. A recent study of 743,948 cases over 25 years identified an elevated risk of developing AD among individuals using non-selective β-AR antagonists, while individuals administered selective β_2_-AR agonists had a lower risk (Hutten et al., 2022). Activation of β_2_-AR by the intranasal route suppressed Tau hyperphosphorylation and the preservation of synaptic integrity within Aβ-treated rat hippocampal neuronal cells (Groo et al., 2025). Furthermore, ablation of the β_2_-AR gene rescued pathological features of senile PS1/APP mice, indicating that β_2_-AR is an attractive target for therapeutic purposes for the alleviation of AD (Wang et al., 2013a).

In this review, we examine the therapeutic prospect of utilizing β_2_-adrenergic pathways for providing neuroprotection against AD. We highlight how activation of β_2_-AR maintains synaptic plasticity, prevents Aβ-mediated deficits, and modulates inflammation, mitochondrial function, and epigenetic mechanisms. Through the synthesis of available evidence, our goal is to shed light on the therapeutic value of β_2_-AR signaling for the treatment of synaptic dysfunction and cognitive decline in AD.

## Search Strategy

This review is completed based on literature identified by searches conducted on PubMed, ScienceDirect, and ClinicalTrials.gov. Targeted search terms included various pertinent issues, including Alzheimer’s disease, amyloid-β, astrocytes, β_2_-adrenergic signaling (synonyms: beta-adrenergic receptor, beta2-adrenergic receptor, b2-adrenergic, and β_2_-AR/β_2_-AR), epigenetic mechanisms, histone modification, neuroinflammation, activity of microglia, synaptic plasticity, long-term potentiation, cognition, enriched environmental exposure, mitochondrial function, activity of microRNAs, and activity of reactive oxygen species. The review of the literature included only publications written in English between 2005 and March 2025. Particular effort was made to select studies examining how β_2_-AR agonists affect synaptic mechanisms and impact AD pathophysiology.

## Enriched Environment and β_2_-Adrenergic Receptor Activation in Alzheimer’s Disease Models

### Enriched environments protect against amyloid-beta-induced synaptic dysfunction

Enriched environments (EE) are experimental settings that provide cognitive and sensory stimulation through greater social interactions, physical exercise, and exposure to novel objects. Numerous studies have shown powerful neuroprotective actions of EE in models of neurodegenerative diseases, including AD (Li et al., 2013; Stozicka et al., 2020; Wei et al., 2020; Liew et al., 2022; Macartney et al., 2022; Mohd Sahini et al., 2024; Nam et al., 2024). In animal models of AD, EE enhances cognitive performance (Stozicka et al., 2020; Mohd Sahini et al., 2024; Nam et al., 2024), stimulates neurogenesis (Ziegler-Waldkirch et al., 2018; Saheb et al., 2023), and prevents Aβ oligomers-mediated synaptic dysfunction (Li et al., 2013; Wei et al., 2020; Mercerón-Martínez et al., 2021). These protective actions of EE are most likely attributed to improvements in synaptic plasticity, as measured through enhanced LTP and LTD, and dendritic spine density of the hippocampus (Artola et al., 2006; Li et al., 2013; Milbocker et al., 2024).

One of the key pathways by which EE can exert its neuroprotective actions is through the modulation of BDNF, an essential regulator of neuronal viability and adaptability of synaptic connections. Experimental evidence is such that exposure to an EE is shown to produce an upregulation of hippocampal expression of BDNF (Aldhshan and Mizuno, 2022; Azman and Zakaria, 2022; Cutuli et al., 2022; Costa et al., 2023), even when Aβ oligomers are present, thus supporting synaptic integrity and cognitive function. EE further enhances the activity of glutamate transporters, e.g., glutamate transporter 1 (Pintori et al., 2024), promoting better glutamate homeostasis, and reducing excitotoxicity, which is characteristic of Aβ-driven synaptic disruption. These, taken together, demonstrate the ability of EE to repress the adverse effects of Aβ oligomers on cognitive function and adaptability of synaptic linkages.

### Impact of enriched environments on Tau pathology

Tau pathology, involving the hyperphosphorylation and assembly of the microtubule-associated tau protein, is a prevalent feature of AD that is highly correlated with the development of the disease and its concomitant loss of cognitive function (Johansson et al., 2021; Ossenkoppele et al., 2021; Leinenga et al., 2024). The neurotoxic actions of tau are speculated to impair synaptic function and neuronal connectivity, adding to the adverse effects of Aβ on neural circuits. EE is shown to have substantial neuroprotective actions opposing tau pathology across preclinical and clinical studies (Lahiani-Cohen et al., 2011; Selvi et al., 2017; Stozicka et al., 2020; Mate et al., 2022). In tauopathy mouse models, EE diminishes the levels of hyperphosphorylated tau, and tau-mediated synaptic impairments (Hu et al., 2013). These actions are mediated, at least partly, by enhancing neuronal activity, and the upregulation of neurotrophic factors such as BDNF, which support neuronal health and plasticity.

Additionally, EE stimulates mechanisms of proteostasis, such as activity of the ubiquitin-proteasome system, and autophagy (Takahashi et al., 2014; Xu et al., 2022, 2023), which enable the removal of pathological tau species. EE is also known to alter the activity of kinases, and phosphatases, targeted for tau phosphorylation. For instance, enriched environments have been linked with lesser activity of glycogen synthase kinase 3 beta (Hu et al., 2013; Llorens-Martin et al., 2013; Scala et al., 2018), a critical tau kinase, which leads to lower levels of hyperphosphorylated tau. These observations highlight the therapeutic value of EE in the modulation of tau pathology, a major cause of neurodegeneration of AD.

### Enriched environments guard against Alzheimer’s disease via β_2_-adrenergic receptor activation

Exposure to novel objects and access to running wheels improve hippocampal-dependent memory in juvenile animals and protect against cognitive decline associated with age or neurodegenerative diseases (Mohd Sahini et al., 2024; Neves et al., 2024). These benefits are partly attributed to EE-induced increases in brain norepinephrine levels and enhancement of β-AR signaling (Escorihuela et al., 1995; Cao et al., 2010). In AD models, EE exposure alters Aβ and other protein levels, yet consistently leads to improved cognitive performance (Li et al., 2013).

**Figure 1:**
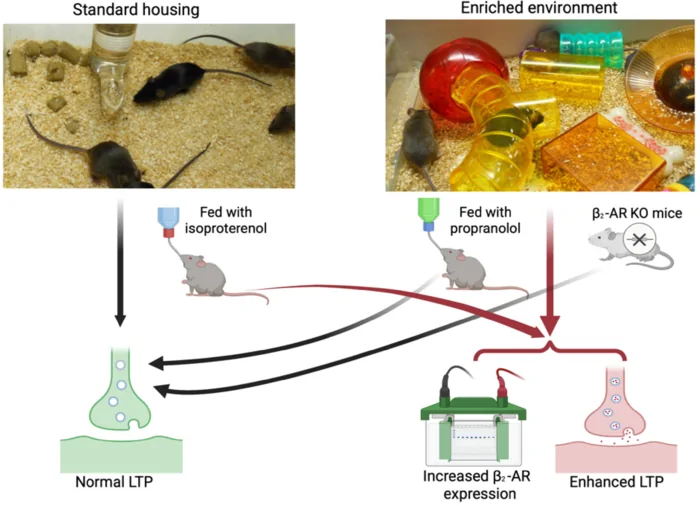
Enriched environment augments hippocampal synaptic plasticity through β-adrenergic signaling. Exposure to enriched environment dramatically enhanced high-frequency stimulation-induced long-term potentiation (LTP) and expression of the β_2_-adrenergic receptors (β_2_-AR) within the hippocampus. The enhancing effect, however, was lost following treatment of the same enriched environment paradigm with the β-adrenergic receptor blocker propranolol, and in β_2_-AR knockout (KO) mice with exposure to the same enriched environment paradigm, suggesting that β_2_-AR signaling must play a role in enriched environment-mediated enhancement of LTP. In contrast, administration of standard-housed mice with the β-AR agonist isoproterenol for 4 weeks replicated the effects of enriched environment, including enhanced LTP, expression of synaptic markers (i.e., postsynaptic density protein 95, glutamate ionotropic receptor NMDA type subunit 1, and glutamate receptor 1), and resistance of LTP to amyloid-beta oligomer impairment. Created with BioRender.com.

We had earlier shown that EE prevents hippocampal synaptic plasticity impairments caused by AD brain-derived Aβ oligomers, an effect mediated by activation of the receptor β_2_-AR (Li et al., 2013). This benefit disappeared in the absence of the receptor, through knockout β_2_-AR, emphasizing the critical role of the receptor (Jin et al., 2023; **Figure 1**). Interestingly, activation of the receptor, β_2_-AR, prevents neurotoxicity in several neurodegenerative diseases, including Down syndrome (Dang et al., 2014; Emili et al., 2020), Parkinson’s disease (Giorgianni et al., 2020; Khidr et al., 2023; Chang et al., 2024; Inchiosa, 2024), amyotrophic lateral sclerosis (Bartus et al., 2016), multiple sclerosis (Araujo et al., 2019), and cerebral ischemia (Lechtenberg et al., 2019; Hu et al., 2024). Clinically, β_2_-AR agonists, such as salmeterol, albuterol, formoterol, fenoterol, and procaterol, are widely prescribed bronchodilators for control of asthma (Ortega, 2014; Cazzola et al., 2019; Slob et al., 2021). It is not known whether activation of the receptor, β_2_-AR, improves learning and memory sufficiently to overcome AD-related deficits.

### Experimental evidence supporting β_2_-adrenergic receptor’s role in enriched environment-induced neuroprotection

A mounting body of evidence highlights the crucial role of β_2_-AR activation for the neuroprotective actions of EE against Aβ-mediated synaptic disruption (Li et al., 2013; Jin et al., 2023; Li, 2023). In AD models of mice, selective β_2_-AR agonists mimic the cognitive and synaptic improvements of EE, reaffirming the receptor’s role during pathological processes for the preservation of neuronal plasticity (Li et al., 2013; Zhou et al., 2013; Saez-Briones et al., 2015). Importantly, activation of β_2_-AR can rescue hippocampal deficits of LTP produced by Aβ oligomers (Li et al., 2013; Li, 2023), reaffirming its critical function for the preservation of synaptic integrity.

Our evidence demonstrated that exposure to EE strengthens hippocampal cAMP signaling, including activation of PKA and cyclic AMP response element-binding protein (CREB), even with Aβ oligomers present (Li et al., 2013). Pharmacological blockade of β_2_-AR eliminates such effects, further affirming its critical function in EE-mediated neuroprotection. Similarly, β_2_-AR knockout mice do not show EE-induced resilience against Aβ-dependent impairments of LTP, providing direct genetic evidence of the receptor’s function (Jin et al., 2023).

Beyond its classical cAMP-mediated neuroprotective role, β_2_-AR activation promotes epigenetic regulation, particularly through microRNAs. EE is shown to increase levels of miRNA-132, an essential regulator of synaptic plasticity and neuronal survival (Wei et al., 2020), and β_2_-AR activation mimics this effect. It rescues Aβ-mediated synaptic impairment through tau-phosphorylation modulation and regulation of glutamate receptor trafficking (Chai et al., 2022). Furthermore, β_2_-AR activation preserves synaptic structures by enhancing α-amino-3-hydroxy-5-methyl-4-isoxazolepropionic acid (AMPA) and N-methyl-D-aspartate (NMDA) receptors trafficking, counteracting tau-related synaptic damage (Patriarchi et al., 2018). These findings highlight the multifaceted role of β_2_-AR signaling in promoting synaptic resilience through both molecular and epigenetic mechanisms.

Activation of β_2_-AR plays a key role in reducing neuroinflammation, a hallmark of AD. Specifically, β_2_-AR activation in microglia suppresses the production of pro-inflammatory cytokines, thereby fostering a neuroprotective environment. Since EE is known to attenuate microglial activation and inflammation (Augusto-Oliveira et al., 2025), these findings suggest that β_2_-AR signaling is a central mediator of anti-inflammatory effects of EE. Moreover, activation of β_2_-AR also promotes autophagy (Deng et al., 2019; Lee et al., 2020; Lazzeri et al., 2021; Wu et al., 2024), a critical process for clearing toxic protein aggregates such as tau. This aligns with previous reports showing that EE enhances autophagic flux (Takahashi et al., 2014; Xu et al., 2022, 2023), supporting the idea that β_2_-AR signaling contributes to EE-induced tau clearance through autophagy activation.

Mitochondrial dysfunction is another characteristic of AD, responsible for oxidative imbalance and energetic deficits of neurons (Meng et al., 2024; D’Alessandro et al., 2025; Spina et al., 2025). Experimental evidence shows that activation of β_2_-AR restores mitochondrial function by promoting PKA-dependent signaling (Dorotea and Ha, 2021; Chai et al., 2022; Chang et al., 2024), supporting mitochondrial biogenesis, and limiting reactive oxygen species (ROS) accumulation. Protective effects are further enhanced by EE, highlighting the cooperative interplay between environmental enrichment and β_2_-AR pathways for neuronal homeostasis maintenance.

**Figure 2:**
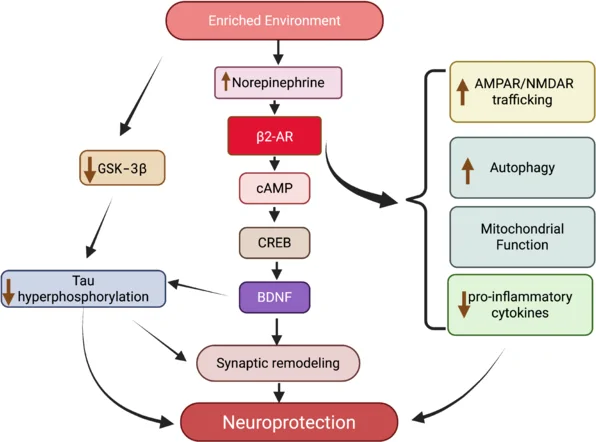
Enriched environments promote neuroprotection through β_2_-adrenergic receptor activation. Exposure to enriched environments increases brain norepinephrine levels, which activate β_2_-AR. This activation enhances synaptic plasticity and reduces tau hyperphosphorylation, in part through upregulation of BDNF. Additionally, β_2_-AR activation promotes the trafficking of glutamatergic AMPA and NMDA receptors, enhances autophagy, restores mitochondrial function, and suppresses the release of pro-inflammatory cytokines. Together, these effects contribute to the neuroprotective actions of enriched environments. Created with BioRender.com. AMPAR: α-Amino-3-hydroxy-5-methyl-4-isoxazolepropionic acid receptor; β_2_-AR: β_2_-adrenergic receptor; BDNF: brain-derived neurotrophic factor; cAMP: cyclic adenosine monophosphate; CREB: cAMP response element-binding protein; GSK-3β: glycogen synthase kinase 3 beta; NMDAR: N-methyl-D-aspartate receptor.

Together, these findings identify β_2_-AR activation as a central mediator of the neuroprotective effects of EE. EE enhances norepinephrine levels, thereby activating β_2_-AR signaling, which promotes synaptic plasticity, regulates glutamatergic receptor trafficking, enhances protein clearance, reduces neuroinflammation, and supports mitochondrial function (**Figure 2**). These multifaceted actions highlight β_2_-AR signaling as a promising therapeutic target for AD. With further research, through the utilization of β_2_-AR pathways, possible mechanisms for reducing AD-linked synaptic deficits and cognitive impairment will emerge, something that will need further evaluation through clinical- and preclinical-level experiments.

## β_2_-Adrenergic Receptor Agonists and Synaptic Plasticity

Synaptic plasticity, in the form of LTP and LTD, is essential for learning, memory, and cognition (Whitlock et al., 2006; Citri and Malenka, 2008; Bazzari and Parri, 2019; Jeong et al., 2021). AD is characterized by dysregulation of these mechanisms, in part initiated by disease-relevant interactions between Aβ oligomers and tau. Repeated exposure to an EE or exposure to new environment may have the potential to elicit hippocampal LTP by activation of β-ARs (Li et al., 2013; O’Dell et al., 2015; Burgos et al., 2019; Ohline and Abraham, 2019). Also, the experiences can result in enlargement of dendritic spines and density (Li et al., 2013). Similarly, β-AR activation by administration of β-AR agonist isoproterenol (ISO) permits a presumably ineffective subthreshold synaptic stimulation to induce vigorous LTP (Havekes et al., 2012; Qian et al., 2012; Ul Haq et al., 2016; Maity et al., 2020). Notably, the enhancement of LTP by ISO in the hippocampal slice from wildtype mice was abrogated when pre-treated with the selective β_2_-AR antagonist ICI-118551 but not influenced by the β_1_-AR antagonist, CGP-20712 (Qian et al., 2012). These findings indicate that activation of β_2_-ARs, rather than β_1_-ARs, considerably rescues hippocampal LTP deficits caused by Aβ oligomers (Wang et al., 2009; Li et al., 2013).

The broadly expressed G protein-coupled receptor, the β_2_-AR, plays an essential role in controlling synaptic plasticity through modulation of several intracellular pathways of signalization. Preclinical research has emphasized the capacity of β_2_-AR agonists for reinforcing LTP and for blocking LTD during synaptic stress, lending strong support for their therapeutic role in AD.

### Evidence for the role of β2-adrenergic receptor agonists in increasing long-term potentiation while blocking long-term depression

The actions of β-AR activation are localized primarily to the synapses. It has been shown that ISO stimulation of β-AR enhances hippocampal LTP through a presynaptic mechanism, as indicated by decreased paired-pulse facilitation of excitatory postsynaptic currents (Wojtowicz et al., 2010). Another study, however, reported that there is no altered paired-pulse facilitation of field excitatory postsynaptic potentials after ISO treatment of hippocampal slices (Papaleonidopoulos and Papatheodoropoulos, 2018). These differences could be attributed to variations in experimental set-up, such as recording location (pyramidal cells of the subiculum as compared to apical dendrites of CA1) or concentrations of ISO employed (2 µM *versus* 1 µM). Together, the evidence supports that activation of β-AR enhances hippocampal LTP through both the presynaptic and postsynaptic mechanisms.

Regarding the impact of EE on short-term plasticity, the majority of studies have found no alteration in paired-pulse facilitation in the hippocampal CA1 or dentate gyrus areas (Li et al., 2013; Ohline and Abraham, 2019). However, paired-pulse inhibition population spike recordings elicited by stimulation of CA1 axons had significant alterations in mice kept under EE conditions (Eckert et al., 2010). These results imply that EE doesn’t directly impact presynaptic excitatory synapses but would rather have more subtle effects on CA1 recurrent inhibition. In a similar fashion, β-AR activation with ISO has also been shown to amplify GABAergic transmission within basket cells in cerebellar areas. The effect is exerted through the activation of β_2_-AR that triggers an intracellular cascade effect involving cAMP, not the β_1_-AR, which does not contribute to this function (Saitow et al., 2000).

Systemic but also experimental studies in rodent models have noted that activation of β_2_-AR increases LTP and counteracts LTD induced by pathological stimuli. For instance, in Aβ oligomers-treated hippocampal slices (which lead to a deficit at the synapse and induce LTD), treatment representing agonism with β_2_-ARs: clenbuterol or formoterol could allow LTP to near-normal levels (Jin et al., 2022, 2023). This rescue of LTP has been observed in both acute Aβ exposure paradigms (McGuckin et al., 2013; Jin et al., 2022, 2023) and in transgenic mouse models of AD, such as 5×FAD and APP/PS1 mice (Zhang et al., 2021), where chronic Aβ accumulation impairs synaptic plasticity.

In addition to rescuing LTP, β_2_-AR activation suppresses aberrant LTD (Larsen et al., 2023). Aβ oligomers have been shown to abnormally facilitate LTD by disrupting glutamate receptor trafficking and promoting the internalization of AMPA and NMDA receptors. Pharmacological activation of β_2_-AR prevents these effects, preserving synaptic receptor density and maintaining synaptic efficacy. The bidirectional regulation of synaptic plasticity by β_2_-AR agonists highlights their potential to stabilize neuronal networks in the context of AD.

### Cellular mechanisms: β_2_-adrenergic receptor in cyclic adenosine monophosphate/protein kinase A signaling and synaptic targets

LTP driven by β-AR signaling is primarily mediated through β_2_-AR activation, which initiates a cAMP-dependent cascade essential for synaptic plasticity. Activation of postsynaptic NMDA receptors elevates intracellular cAMP, leading to PKA activation and downstream signaling through effectors such as extracellular signal-regulated kinase (ERK), both critical for LTP induction and maintenance (Lin et al., 2003; Tenorio et al., 2010). PKA phosphorylates the AMPA receptor subunit GluA1 at Ser845, enhancing synaptic strength by promoting AMPA receptor insertion and reducing internalization (Man et al., 2007). Notably, β_2_-AR activation also counters Aβ-induced AMPA receptor internalization by stabilizing receptor-associated scaffolding proteins, thereby preserving synaptic integrity (Midorikawa et al., 2024; Prinkey et al., 2024). These protective effects are likely mediated by the cAMP/PKA pathway, which facilitates AMPA receptor trafficking, supports LTP, and reduces vulnerability to LTD. Additionally, β_2_-AR signaling modulates NMDA receptor function by enhancing phosphorylation of GluN2B subunit, promoting calcium influx required for calcium-dependent LTP (Murphy et al., 2014; Larsen et al., 2023). Thus, β_2_-AR agonists can mitigate Aβ-induced synaptic dysfunction through coordinated regulation of AMPA and NMDA receptor signaling pathways.

Activation of β_2_-ARs facilitates hippocampal LTP could be by modulating CaMKII signaling. Isoproterenol, a β-AR agonist, shifts the movement of CaMKII from inhibitory synapses to excitatory ones, thereby facilitating the induction of LTP even in conditions that would normally promote LTD. This is scaffolded by non-ionotropic NMDA receptor GluN2B subunits, highlighting the structural and not merely ionotropic functionality of NMDA receptors (Larsen et al., 2023). Further, the β_2_-AR-induced LTP does not necessarily depend on calcium influx through NMDA receptors but rather through L-type voltage-gated calcium channels. It enhances GluA1 subunit phosphorylation at Ser845, promoting AMPA receptor trafficking and synaptic reinforcement (Babushkina and Manahan-Vaughan, 2022; Larsen et al., 2023).

β_2_-ARs play frequency-specific roles in regulating hippocampal LTD. High-frequency stimulation of the locus coeruleus (LC) at Hz, which mimics elevated arousal states, induces LTD in a β_2_-ARs-dependent manner. In contrast, LTD induced by low-frequency stimulation (2–10 Hz) representing tonic LC activity is mediated by D1/D5 dopaminergic receptors rather than β_2_-ARs (Babushkina and Manahan-Vaughan, 2022). These data are consistent with the notion that β_2_-ARs promote synaptic plasticity under conditions of heightened arousal or focused attention, facilitating rapid and robust memory encoding. This suggests that β_2_-ARs act as modulators of hippocampal plasticity, enabling a flexible switch between LTD and LTP, in part through the regulation of ionotropic receptor trafficking at postsynaptic sites. Such adaptability is essential for meeting dynamic cognitive demands and supporting memory formation.

Another important downstream β_2_-AR target is the synaptic plasticity transcription factor CREB (Tanaka et al., 2020; Hajiaghayi et al., 2024). Phosphorylation of CREB by PKA enhances its activity, triggering the transcription of synaptic plasticity genes, including BDNF (Safi et al., 2022). BDNF is crucial for synaptic plasticity and promotes the stabilization of receptors and the formation of new dendritic spines, processes which are impaired in AD. Importantly, β_2_-AR agonists are known to restore the phosphorylation form of CREB and BDNF expression in the hippocampal neurons treated with Aβ, strengthening their role in synaptic recovery (Counts and Mufson, 2010).

### Pharmacological experiments with β_2_-adrenergic receptor agonists evidence

Using selective β_2_-AR agonists for spaced repetition learning in pharmacology demonstrates strong support for the ability to foster synaptic plasticity. The highly selective β_2_-AR agonist Clenbuterol has been proven capable of recovering hippocampal slice LTP after Aβ oligomer exposure. It is also dose-dependent and blocked by β_2_-AR antagonists, proving the specific effect of Clenbuterol. Sufficient evidence has also been observed with Salmeterol and Formoterol, which are long-acting β_2_-AR agonists used in a clinician’s setting for the treatment of asthma, highlighting the importance these cases hold.

The therapeutic potential of β_2_-AR agonists comes to light in *in vivo* experiments. Clenbuterol administration over extended periods in APP/PS1 mice led to improved performance in memory-related behavioral tests alongside observed increases in dendritic branching and spine growth, while hippocampal synaptic loss was witnessed, too (Chai et al., 2016, 2017). Accompanying these effects were declines in the levels of hyperphosphorylated tau and neuroinflammation responses, suggesting additional action, alongside the synaptic control, that β_2_-AR agonists have.

Additionally, β_2_-AR agonists have also proven to be viable in reversing tau pathology-mediated synaptic impairments. In AD model mice, administration of clenbuterol has been shown to decrease tau hyperphosphorylation and aggregation, thus preserving both synaptic form and function (Chai et al., 2022). This implies that activation of β_2_-AR can reverse amyloid- and tau-mediated synaptic dysfunction in AD.

## Immunomodulatory Effects of β_2_-Adrenergic Receptor Activation in Alzheimer’s Disease

The β_2_-AR is involved not only in neurotransmission but also has immunomodulatory activity. Such effects are of increasing interests within the framework of AD, where the process of neuroinflammation is central to the development of the disease. The activation of β_2_-AR has been found to be able to modulate the process of neuroinflammation by regulating the behavior of microglia (Damo et al., 2023; Le et al., 2023) and astrocytes (Hosseindoost et al., 2020; Ingiosi and Frank, 2022), and regulating cytokine release (Farooq et al., 2023; Zhang and Shen, 2024). Such results demonstrate the therapeutic potential of β_2_-AR agonists to counteract the process of neuroinflammation related to AD.

### Loss of noradrenergic tone and β_2_-adrenergic receptor dysfunction in Alzheimer’s disease

The LC, the main cerebral norepinephrine source, is progressively impaired with normal aging and is one of the first areas affected in AD (Theofilas et al., 2017; Matchett et al., 2021; Mercan and Heneka, 2022; Li, 2023). Noradrenergic tone is more fully disrupted in AD and underlies disease pathology. Experimental LC lesion or pharmacological β-adrenergic receptor blockade has been shown to increase neuroinflammation and cognitive and behavioral impairments in AD mouse models (Ardestani et al., 2017; Evans et al., 2020).

Microglia, the immune cells of the brain, are especially rich in β_2_-AR, expressing them at significantly higher levels than any other cell types in the central nervous system (CNS) (Bennett et al., 2016; Jensen et al., 2016; Chhatar and Lal, 2021). This implies that microglia could be specifically designed to sense NE signaling through β_2_-ARs. In fact, more recent research using chemogenetic inhibition of LC activity, pharmacological blockage of β-adrenergic receptors, or conditional deletion of Adrb1 or Adrb2 in microglia, depicted that inhibition of β_2_-AR signaling in microglia accelerates amyloid plaque formation and neuritic damage. Conversely, augmentation of microglial β_2_-AR activity inhibits amyloid pathology, demonstrating the protective nature of this signaling pathway (Evans et al., 2024; Le et al., 2025).

Ironically, Aβ peptides directly interact with β_2_-ARs and interfere with their function in various ways. Aβ interaction on β_2_-AR leads to hyperactivation of PKA, enhancing the activity of AMPA receptors and calcium influx — processes leading to excitotoxicity in neurons and cell death in AD transgenic mice (Wang et al., 2010). Aβ is also responsible for β_2_-AR internalization and degradation and eliciting blunted adrenergic and glutamatergic synaptic transmission within the key brain areas such as the prefrontal cortex, a sign of neurodegenerative disorders (Wang et al., 2011).

Moreover, internalization of β_2_-ARs by Aβ also activates the β-arrestin pathway, which has been found to induce Aβ production and promote AD pathology (Kee et al., 2024). Moreover, Aβ binding to β_2_-AR has been shown to stimulate the PKA–JNK signaling cascade leading to the hyperphosphorylation of tau at disease-relevant sites such as Ser214, Ser262, and Thr181, further implicating β_2_-AR dysfunction in the pathological neuropathology of AD (Wang et al., 2013a; Wu et al., 2020).

**Figure 3:**
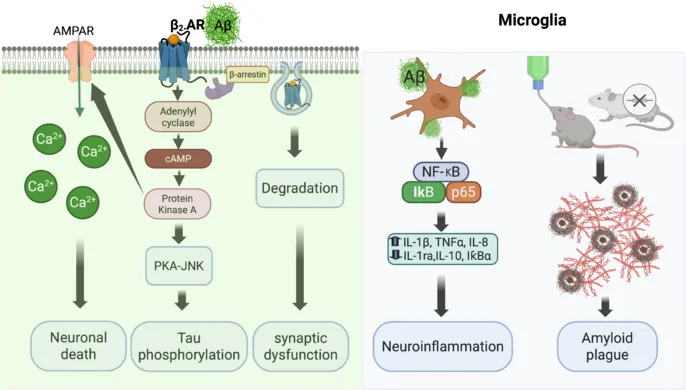
Dysfunction of β_2_-ARs in neurons and microglia accelerates Alzheimer’s disease pathology. In neurons, Aβ binding to β_2_-ARs activates AMPARs via PKA signaling, leading to excessive calcium influx and neuronal death. Concurrently, activation of the PKA–JNK pathway increases tau hyperphosphorylation. Endocytosis of β_2_-ARs disrupts both adrenergic and glutamatergic signaling, resulting in impaired synaptic plasticity. In microglia, Aβ interaction with β_2_-ARs triggers the release of proinflammatory cytokines, contributing to neuroinflammation. Moreover, chemogenetic inhibition of locus coeruleus activity, pharmacological blockade of β-adrenergic receptors, or microglia-specific deletion of the Adrb2 gene significantly accelerates amyloid plaque formation. Created with BioRender.com. AMPAR: α-Amino-3-hydroxy-5-methyl-4-isoxazolepropionic acid receptor; Aβ: amyloid-beta; cAMP: cyclic adenosine monophosphate; IL: interleukin; IL-1ra: interleukin-1 receptor antagonist; IκB: inhibitor of kappa B; JNK: c-Jun N-terminal kinase; NF-κB: nuclear factor kappa B; PKA: protein kinase A; TNFα: tumor necrosis factor alpha; β_2_-AR: β_2_-adrenergic receptor.

LC degeneration and norepinephrine tone loss thus lead to neuroinflammation and cognitive decline. Protective β_2_-ARs on microglia regulate amyloid pathology, while Aβ peptides interfere with this through triggering β_2_-AR internalization, causing harmful signaling cascades, and augmenting tau phosphorylation and neuronal damage (**Figure 3**). These results highlight the bivalent nature of Aβ action on β_2_-AR signaling and its involvement in AD progression.

### Microglial and astrocytic function regulation

Resident CNS immune cells, the microglial cells, play a crucial role in maintaining neural homeostasis and are involved both in monitoring and keeping the brain healthy. In AD, they become pro-inflammatory at frequent intervals, aggravating neuronal destruction by secreting cytokines such as tumor necrosis factor alpha (TNF-α) and interleukin (IL)-1β (Sharma and Flood, 2018). Stimulation of β_2_-ARs has been found to dampen this pro-inflammatory process through nuclear factor kappa B (NF-κB) inhibition, limiting cytokine release and favoring an anti-inflammatory, protective phenotype (Heneka et al., 2010). The stimulation of β_2_-ARs has been found to increase Aβ plaque removal by microglia, an essential pathological hallmark of AD, thereby lowering neuronal toxicity and synaptic impairment (Xu et al., 2018; Jin et al., 2023).

The activation of microglia is an important factor in the development of AD, while signaling through β_2_-AR is important for maintaining homeostasis regarding the protective and detrimental functions of microglia. We found out that in human Aβ oligomer-induced AD model mice, EE, particularly through action of β_2_-AR, can inhibit microglial-mediated neuroinflammation (Xu et al., 2016, 2018). Similarly, activation of β-ARs by administering ISO to APP^NLGF^ mice significantly prevents Aβ-induced synaptic dysfunction and reduces microglial inflammation (Jin et al., 2025). It has also been demonstrated that activation of β_2_-AR suppresses the neuroinflammatory chronic state by reducing TNF-α, IL-6, and IL-1β excretion, utilizing anti-inflammatory mechanisms through the suppression of NF-κB and other inflammatory pathways (Sharma et al., 2019; Damo et al., 2023). However, signaling through β_2_-AR promotes excretion of inhibitory neurotrophic factors such as BDNF (Juric et al., 2008; Han et al., 2019), which is critical for fostering neural cell survival and sustaining synaptic plasticity. Also, β_2_-AR stimulation enhances the activity of autophagy in microglial cells, with the result of dismantling and eradicating many Aβ peptides (Wu et al., 2024).

Another important type of glial cells, which are astrocytes, may also display functional changes as a result of β_2_-AR activation. Depending on their activation states, these cells participate in neuroprotection and contribute to the process of neurodegeneration (Rodriguez-Giraldo et al., 2022; Lopez Couselo et al., 2024). Stimulation of the β_2_-AR is reported to reduce the release of ROS and pro-inflammatory cytokines from these cells, thereby enhancing the preservation of blood–brain barrier (BBB) and the decrease of neuroinflammatory cascades (Kelly et al., 2017). More importantly, astrocytic rather than neuronal β_2_-ARs in the hippocampus play a crucial role in consolidation of fear-based contextual memory (Gao et al., 2016). β_2_-ARs also mediate training-induced astrocytic lactate release, a process critical for long-term memory establishment, and with molecular adaptations to it. It points towards the potential metabolic function of astrocytic β_2_-ARs as a future drug target against neurodegenerative and stress disorders.

Further, astrocytic β_2_-AR signaling also results in enhanced release of anti-inflammatory mediators such as transforming growth factor-beta, bolstering its neuroprotective role. The role of β_2_-AR in modulating both inflammation and excitotoxicity concurrently places it at the forefront as a therapeutic target for AD treatment.

### Cytokine regulation and systemic effects

The adjustment of cytokines by β_2_-AR agonists plays an important role in bringing immune homeostasis (Sharma et al., 2019; Damo et al., 2023). β_2_-AR activation, for instance, increases IL-10 production and encourages the conversion of microglia and macrophages to an M2 reparative phenotype. This is important since microglial and macrophages in the M2 phenotype participate in cellular debris and misfolded proteins removal and mitigate apoptotic damage via phagocytosis. Another one is the reduction of pro-inflammatory mediators such as IL-6 and TNF-α, which serve to reduce systemic inflammation along with the other inflammatory cascade targeting the CNS (Mohammadpour et al., 2019; Jin et al., 2023). Of note, apart from more localized functions in the CNS regarding actions from β_2_-AR stimulation, greater peripheral immunomodulatory functions are noted. In general, the actions of β_2_-AR result in the suppression of pro-inflammatory cytokines such as IL-1β, TNF-α, and IL-6, and a response by increasing anti-inflammatory mediators such as IL-10 (Sharma et al., 2019; Evans et al., 2024). This central statistical orthodoxy to modulate the provocative response would succeed in addressing excessive immune responses that become disturbing and unfettered in disease processes presented as chronic inflammation, sepsis, autoimmunity, and systemic reactions that pose such disruptions and undesirable effects. Moreover, the β_2_-AR signaling effect appears to modulate γ-secretase activity as an enzyme linked to the production Aβ (Ni et al., 2006). Therefore, perhaps indirectly, β_2_-AR agonists not only modulate a regulatory immune action but also may keep a lower Aβ accumulation, at the very least, providing the unique therapeutic benefit of dual effects: reducing neuroinflammation and limiting the plaque accumulation.

Following earlier cited conclusions, the activity of β_2_-AR will reduce the inflammatory mediators (IL-1β, IL-8, and TNF-α) and increase the level of anti-inflammatory factors, IL-10, IκBα, and IL-1 receptor antagonist (IL-1ra) in numerous immune competent cells in the nervous system. This is further evidence to support the potential of β_2_-AR modulation as a therapeutic approach to neuroinflammatory and neurodegenerative disorders (Sharma and Flood, 2018; O’Neill et al., 2020; Tanaka et al., 2020; Jurgens et al., 2024; Zhang and Shen, 2024; Ford et al., 2025).

### Immunomodulatory effects of β_2_-adrenergic receptor activation

The attention garnered using β_2_-ARs as immune modulators is well warranted, particularly in the context of treating inflammatory and autoimmune diseases. β_2_-ARs have shown promising roles in maintaining immune system balance by modulating cytokine release, immune cell function, and inflammation across various disease processes.

#### Sympathetic-immune interplay

The involvement of β_2_-AR on immune cells is extensive, including T and B lymphocytes, dendritic cells, and macrophages. This vast representation provides evidence that β_2_-ARs are essential components of the sympathetic nervous system that modifies immune responses. These receptors interact with norepinephrine, which is what connects the sympathetic nervous system and immune systems. On stimulation, β_2_-AR pathways most commonly stimulate the cAMP/PKA signaling pathway, which is important in balancing the immune response to infection and injury (Lorton and Bellinger, 2015; Mohammadpour et al., 2019; Jin et al., 2023). β_2_-AR stimulation regulates the pro-inflammatory T-helper 1 (Th1) response and promotes the anti-inflammatory T-helper 2 (Th2). This diverse change in immune response will be effective in combating autoimmune diseases, but will increase the risk of developing infections (Lorton and Bellinger, 2015; Jin et al., 2023).

### Context-dependent signaling mediates variability

New emerging evidence suggests that β_2_-AR signaling is context-dependent and cannot be generalized across all immune cells and situations. In some cases, the β_2_-AR can switch from the “natural” cAMP-PKA pathway to the mitogen-activated protein kinase cascade, which can lead to different, context-dependent immunoregulatory effects. It is plausible that this may explain the variability seen in β_2_-AR-mediated immunity in the various diseases, and triggers further studies are warranted into the precise mechanisms governing this transition (Lorton and Bellinger, 2015).

### Therapeutic implications and challenges

β_2_-AR activation has far-reaching immunomodulatory effects that make it a potential therapeutic avenue in types of autoimmunity, neuroinflammation, and for cancer immunotherapy, yet drug side effects and therapeutic indexing present many hurdles in proper translation. In AD, β_2_-AR agonists can provide a neuroprotective effect through modulating astrocyte and microglial activity, downregulating inflammatory cytokines, and increasing Aβ clearance. Yet these benefits come with tremendous challenges: optimizing receptor targeting, minimizing off-target effects, and unraveling signaling dynamics relevant to disease. Additional research must be performed to optimize β_2_-AR-based therapeutic interventions and incorporate them into effective, extensive treatment regimens for inflammatory and neurodegenerative disorders.

## Epigenetic Mechanisms Underlying β_2_-Adrenergic Receptor Activation in Alzheimer’s disease

β_2_-AR epigenetic regulation of activation is a novel area of AD research. β_2_-ARs belong to the G-protein-coupled receptor family with significant influences on synaptic plasticity, neuroinflammation, and neuroprotection. Activation has been associated with therapeutic transcriptional changes through DNA methylation, histone remodeling, and miRNA regulation that together reduce AD pathology by enhancing neuroprotective gene expression while inhibiting disease-supporting entities (Maity et al., 2016).

### Epigenetic mechanisms of β_2_-adrenergic receptor activation

#### Histone modifications

AD pathogenesis mainly involves a novel and dynamic regulation of histone acetylation and deacetylation, which leads to changes in the pattern of gene expression. β_2_-ARs activation can increase histone acetylation for promoting the expression of neuroprotective genes. This is mediated through recruiting histone acetyltransferase, such as CREB-binding protein, as one form of supportive synaptic plasticity and cognition (Xu et al., 2015). β_2_-AR signaling simultaneously inhibits HDAC2/3 overexpression, considered to be a critical regulator of synaptic functioning (Wei et al., 2020; Jin et al., 2023). Indeed, inhibition of these deacetylases, for example, rescues LTP in the hippocampus (Wei et al., 2020; Jin et al., 2023), which is acutely required for learning and memory storage. Phylogenetically, cAMP-PKA-dependent phosphorylation is one of the key mechanisms by which β_2_-AR signaling performs its epigenetic actions that suppresses HDAC2/3 function, leading to H3 acetylation at promoters of synaptic plasticity-driven genes such as BDNF, Arc, and PSD95 (Shivakumar et al., 2020; Katayama et al., 2022; Wu, 2025). These changes in chromatin remodeling result in long-term enhancement of the resilience of long-distance synaptic function against Aβ toxicity, thus justifying the neuroprotective role of β_2_-AR in AD.

Furthermore, histone H3 lysine 27 acetylation (H3K27ac) at the core regulatory regions is influenced by β_2_-AR activation (Mittal et al., 2017). A fascinating twist here is that β_1_-AR agonists have been shown to lower H3K27ac levels at certain promoter and enhancer sites, but the β-blocker propranolol acts against itself in its effect to increase acetylation at regulatory regions (Li et al., 2016). This indicates that β_2_-AR signaling likely acts in a biphasic manner under the context of histone modifications, pharmacologically dependent. Because HDAC2/3 inhibition was previously reported to enhance synaptic plasticity in AD models, an epigenetic targeting against the β_2_-AR signaling may be a promising therapeutic rationale for restoring Aβ-induced synaptic dysfunction.

#### DNA methylation

Aberrant patterns of DNA methylation are highly implicated in AD pathogenesis, frequently silencing genes important for neuronal integrity. Activation of β_2_-AR has been shown to reverse hypermethylation of neuroprotective genes and restore their expression, enhancing cognitive resilience.

A recent study identified ADRB2, the gene coding for β_2_-AR, to be hypermethylated in models of AD, and this correlates with repressed β_2_-AR expression. Epigenetic repression is responsible for blocking PI3K/Akt signaling, a survival pathway essential to neurons, and further triggering pro-apoptotic cascades such as Bcl-2/BAX and p53 signaling (Yang et al., 2019). Experimental data by bisulfite sequencing PCR and methylation profiling validated ADRB2 hypermethylation as the determiner of decreased β_2_-AR activity and that targeting the same could restore protective adrenergic signaling in AD.

#### MicroRNA regulation

The activation of β_2_-AR is a critical event in the regulation of the expression of specific miRNAs that are key regulators of synaptic function and neuroprotection in AD. One of the most well-characterized is miRNA-132, which is essential for synaptic maintenance, neuroprotection, and tau regulation (Zhang and Bian, 2021; Zeng et al., 2022). MiRNA-132 dysfunction has been well linked to AD pathophysiology, including synaptic dysfunction and neurodegeneration (Bahlakeh et al., 2021; Kouhnavardi et al., 2023).

The CREB-dependent transcription of miRNA-132 is upregulated by β_2_-AR activation (Hansen et al., 2013; Yan et al., 2021). Inhibition of glycogen synthase kinase 3 beta and CDK5 enhances neuroprotectors, reducing tau hyperphosphorylation by inhibiting the tau kinase pathways (Liu et al., 2017). Besides, miRNA-132 may protect against Aβ-induced pathology by alleviating oxidative stress and mitochondrial dysfunction (Di Emidio et al., 2014), stabilizing dendritic spine integrity (Park et al., 2011) and synaptic receptor trafficking—all of these are critical for preservation of synaptic plasticity. Considering its maintenance of neuronal function, loss of miRNA-132 is an auto hallmark of AD pathology.

β_2_-AR signaling upregulates miRNA-132 (Park et al., 2011; Jin et al., 2023) and this upregulation decreases hyperphosphorylation tau and toxicity associated with both Aβ (Chai et al., 2022). β_2_-AR stimulation restores miRNA-132 expression, which may be an interesting therapeutic option to prevent Aβ-induced synaptic dysfunction and neurodegeneration. These actions target multiple elements of AD pathologies, such as hyperphosphorylation at tau in axon cable, mitochondrial damage, and synaptic loss. Based on this hypothesis, β_2_-AR-mediated miRNA-132 upregulation could be an effective intervention that enhances neuronal resilience and delays disease progression.

### β_2_-Adrenergic receptor-based epigenetic modulation of neuroinflammation

Chronic neuroinflammation, caused by excessive activation of microglia and astrocytes in AD, drives extensive neurodegeneration. Acute and chronic exposure to β_2_-AR down-regulates inflammatory pathways through epigenetic mechanisms. Histone modifications control the repression of pro-inflammatory cytokine production by β_2_-AR stimulation β_2_-AR activation increases histone acetylation at anti-inflammatory gene promoters particularly (Frost et al., 2004). It results in the suppression of NF-κB responsive inflammatory cascade and thereby minimizes the harmful aspects of reactive gliosis.

New evidence for chromatin remodeling of the β_2_-AR gene promoter reveals more about immune regulation. Experiments have proven that H3K9 and H3K27 demethylation and elevated CpG methylation suppress β_2_-AR expression in pro-inflammatory conditions. In contrast, histone acetylation (H3/H4) and H3K4 methylation, features of active chromatin marks, augment β_2_-AR gene expression in anti-inflammatory conditions (McAlees et al., 2011). These observations imply that pharmacological modulation of β_2_-AR expression with epigenetic treatments may alleviate neuroinflammation in AD.

Overall, epigenetic control of β_2_-AR signaling comes across as a master mechanistic platform incorporating neuroprotection, synaptic transmission, and inflammation in AD. With advancing technology, the identification of targeted β_2_-AR-directed therapies, perhaps with the use of β_2_-AR agonists plus epigenetic drugs, might provide a new and more successful approach to offset AD pathology. Further research should be conducted to refine pharmacological strategies maximizing these epigenetic treatments to implement them in the clinic.

## β_2_-Adrenergic Receptor Regulates Mitochondrial Function

Mitochondrial dysfunction is an important very early AD biomarker that could impact synaptic function, amplify oxidative stress, and neurotoxic damage (Sharma et al., 2021). Recently, β_2_-ARs have been recognized as key regulators of mitochondrial function, influencing biogenesis, dynamics, and inter-organelle communication (Miura et al., 2007; Arif et al., 2019; Vekaria et al., 2020). β_2_-ARs activation was found to enhance energy metabolism, induce mitochondrial biogenesis via peroxisome proliferator-activated receptor-gamma coactivator-1α (PGC-1α) (Miura et al., 2007; Arif et al., 2019), and restore the mitochondrial fission-fusion balance (Vekaria et al., 2020). Moreover, β_2_-AR signaling is essential in driving endoplasmic reticulum (ER)-mitochondria interaction, which is critical for maintenance of calcium homeostasis and adenosine triphosphate (ATP) production (Lim et al., 2021). All these processes highlight β_2_-ARs as drug targets to reduce mitochondrial dysfunction in AD.

### β_2_-Adrenergic receptor activation and mitochondrial biogenesis

Mitochondrial dysfunction is characteristic of AD, including impaired activity of cytochrome c oxidase (complex IV), increased ROS production, and oxidative tissue damage (Sharma et al., 2021). Aβ peptides further potentiate this dysfunction by inserting themselves into mitochondrial membranes and inhibiting enzymatic activity, leading to an increase of local oxidative stress that disrupts synaptic function (Lustbader et al., 2004). Since mitochondria are centrally involved in neuronal energy metabolism, ameliorating mitochondrial deficiencies represents a promising therapeutic approach for AD.

Additionally, β_2_-ARs activation enhances mitochondrial biogenesis, primarily via a PKA-CREB-dependent pathway that activates PGC-1α, a critical regulator of the mitochondrial energy metabolism (Miura et al., 2007; Arif et al., 2019). PGC-1α upregulates key oxidative phosphorylation (OXPHOS) genes, including NDUFS1 and COX5A, and controls mitochondrial dynamics to balance fusion and fission (Jia et al., 2025). β_2_-AR agonists reverse ATP production, increase electron transport chain activity, and inhibit oxidative stress in neurons treated with Aβ, highlighting their role in maintaining neuronal bioenergetics (Wills et al., 2012).

Even though both the short isoform of β_2_-AR and the two α_1_ subtypes can be activated by β_2_-AR selective agonists, the effects of these agonists on mitochondrial biogenesis seem to occur through different activation pathways. The β_2_-AR agonist clenbuterol, but not β_1_- or β_3_-AR agonists, raises skeletal muscle levels of PGC-1α mRNA, an effect blocked by propranolol and in β-AR knockout models (Scholpa et al., 2019). Moreover, PGC-1α expression responsive to β_2_-AR signaling is partially retained during exercise, hinting at the contribution of β_2_-AR signaling in mitochondrial regulation. A second β_2_-AR agonist, formoterol, activates mitochondrial function through both the cAMP-PKA and a new G-protein βγ subunits (Gβγ)-Akt-endothelial nitric oxide synthase (eNOS)-soluble guanylate cyclase (sGC) pathway (Vekaria et al., 2020). This pathway stimulates mitochondrial respiration, *NDUFS1* gene expression, and global mitochondrial biogenesis, whose actions are inhibited by Gβγ, Akt, NOS, and sGC antagonists. Structural docking studies demonstrate that formoterol interacts preferentially with β_2_-AR residues V114 and F193, locking a receptor conformation favoring downstream mitochondrial signaling (Cameron et al., 2017).

Preclinical models also increase the therapeutic potency of β_2_-AR agonists on mitochondrial protection. Formoterol administration was found to cause PGC-1α, complex I, III, and V electron transport chain proteins and markers of mitochondrial biogenesis, along with decreasing apoptosis and improving mitochondrial function (Arif et al., 2019). These results demonstrate the potential of β_2_-AR signaling to reverse Aβ-induced mitochondrial pathology and hence favor β_2_-AR agonists as an option to reverse neuronal bioenergetics and resilience in AD.

### β_2_-Adrenergic receptor modulation of mitochondrial dynamics

Mitochondria undergo repetitive fusion and fission, which are vital for maintaining cellular energy balance, adapting to metabolic demands, and regulating apoptosis (Cho et al., 2010). Mitochondrial dynamism dysregulation plays a role in various neurodegenerative disorders, such as Parkinson’s disease and AD.

The capacity of formoterol to promote mitochondrial function by restoring the equilibrium between fusion and fission and mitochondrial transport has been established by studies. This β_2_-AR agonist was observed to increase mitochondrial DNA copy number, decrease ROS levels, and restore complex III activity but not significantly affect ATP-linked respiration. The mechanism of regulation is through β_2_-AR-mediated modulation of ERK and Akt signaling, with formoterol normalizing ERK signaling and inhibiting overactivation of Akt (Chang et al., 2024). These findings suggest that β_2_-AR signaling is a critical component of mitochondrial homeostasis and may be a promising candidate for neuroprotection in AD and related disorders (Cameron et al., 2019; Cleveland et al., 2020; Vekaria et al., 2020).

### β_2_-Adrenergic receptor regulation of endoplasmic reticulum-mitochondria interactions

G-protein-coupled receptors comprise a large family of receptors in the human genome and are key players in the sensing of extracellular stimuli and signaling cascade via heterotrimeric G proteins. Because G protein-coupled receptors preferentially couple to specific G-proteins upon activation, specific signaling pathways are initiated. The β_2_-AR primarily signals through the Gαs-cAMP pathway, activating downstream effectors including PKA and exchange protein directly activated by cAMP. Besides its classical role in cAMP signaling, β_2_-AR activation by promoting the activation of ER-mitochondria crosstalk, a critical circuit for the maintenance of homeostasis and control of metabolism, has recently been demonstrated (Lim et al., 2021).

β_2_-AR possesses one of the mechanisms for enhancing ER-mitochondria crosstalk by regulating the transfer of calcium. Upon agonist binding to β_2_-ARs, actin polymerization is facilitated via CDC42 activation, leading to ER-mitochondria contact sites formation. Mitochondrial-associated ER membranes facilitate increased calcium influx into mitochondria by inhibiting inositol 1,4,5-trisphosphate receptors on the ER, and mitochondrial calcium uniporter channels are promoted, which promotes ATP synthesis and neuronal energy needs (Lim et al., 2021). Furthermore, β_2_-AR-enhanced modulation of mitochondrial calcium homeostasis decreases ROS accumulation and hence potentially rescues against oxidative stress-related damage in neurodegeneration (Bravo et al., 2011; Lim et al., 2021).

Other ER-mitochondria tethering dysregulations have been described in AD as well, supporting synaptic dysfunction and metabolic dysregulation. In pathological conditions, β_2_-AR activation improves metabolic balance and promotes neuronal resilience, possibly due to enhanced ER-mitochondria contacts. These results define a new role of β_2_-AR signaling in sustaining ER-mitochondria communication, which has potential therapeutic relevance for its contribution to AD pathogenesis and therapy.

The β_2_-AR signaling is known to modify mitochondrial function through various mechanisms, including biogenesis, dynamic equilibrium, and inter-organellar crosstalk. Considering the key role of mitochondria in neurodegenerative diseases, a pathway targeting of β_2_-AR is a potential therapeutic approach for AD. However, several challenges can be listed, including the foray of selective agonists that will amplify beneficial signaling without leading to adverse cardiovascular consequences. Further studies need to be aimed at clarifying the precise molecular mechanisms involved in β_2_-AR-mediated mitochondrial regulation and validating the results in clinically relevant models for AD.

## Comparison with Other Adrenergic Receptor Subtypes

ARs, including α- and β-ARs, are G-protein-coupled receptors that regulate cognitive function, neurovascular coupling, and inflammation by responding to norepinephrine and epinephrine. In AD, among others, the early degeneration of the locus coeruleus, the main supplier of norepinephrine, damages this system, causing synaptic and metabolic dysfunction. ARs are widely distributed throughout the central nervous system and play key roles in neuroprotection (Beardmore et al., 2021; Malatt and Tagliati, 2022; Li, 2023; Nikolenko et al., 2024). Of the β-AR subtypes, β_2_-ARs have emerged as an attractive therapeutic target due to their role in the modulation of synaptic plasticity and the regulation of neuroinflammation (Hutten et al., 2022). In the case of other adrenergic subtypes, such as β_1_-AR, β_3_-AR, and α-ARs, though they are involved in various pathophysiological processes, their specific roles and potential for therapeutic intervention in AD are less clear than for β_2_-AR activation.

### β_1_-Adrenergic receptors

β_1_-adrenergic receptors (β_1_-ARs) have drawn attention as therapeutic targets in AD due to their profound effects on cognitive processes, particularly those pertaining to social learning and memory processing. Activation of β_1_-ARs in the medial amygdala enhances the processing of social information that is otherwise impaired in AD. Most importantly, xamoterol-like highly selective β_1_-AR agonists save AD social recognition deficit in a profile of PKA/phospho-CREB signal pathway activation that is most critical for the synaptic plasticity mechanism (Coutellier et al., 2014).

Apart from cognitive impacts, β_1_-AR modulation is also seen to affect neuropathology related to AD. Repeated dosing with xamoterol in 5×FAD mice results in decreased Aβ and tau pathology and lower neuroinflammatory markers (Ardestani et al., 2017). Notably, the β_1_-AR antagonist nebivolol decreased brain content of Aβ in Tg2576 mice but was without effect on cognitive function (Wang et al., 2013b). These results indicate that β_1_-AR activation not only could prevent neuronal loss and AD pathology but also reverse cognitive and social functions, and therefore it is an interesting area to pursue.

In contrast, genetic polymorphisms of the β_1_-AR and G protein beta 3 subunit are potential risk factors for AD development that have been identified (Bullido et al., 2004). These polymorphisms affect adrenergic signaling through altered cAMP levels, mitogen-activated protein kinase activation, and amyloid precursor protein (APP) processing in human cell lines (Bullido et al., 2004). These alterations could be inducing AD pathology, and this would point toward the genetic association of β_1_-AR signaling and risk for disease. Knowing how these genetic elements affect AD development can open new paths for precision medicine strategies targeting adrenergic pathways.

### β_3_-Adrenergic receptors

Of the three subtypes of β-adrenergic receptor (β-AR), β_3_-adrenergic receptors (β_3_-ARs) are most linked to metabolic control in adipose tissue, a key energy depot that adds to systemic glucose, lipid, and energy homeostasis. Worth noting, adipose tissue gradually changes remarkably with aging (Emorine et al., 1989; Mund and Frishman, 2013). In addition to metabolism, β_3_-ARs have been involved in neural function. A newly engineered ligand-directed β_3_-AR fluorescent probe, β_3_-ARP, has shown high specificity for β_3_-AR binding in primary neuronal cells (Liu et al., 2024). This instrument allows real-time observation of lipid droplet accumulation, a process associated with β_3_-AR dysfunction in neurological disease, and thus represents a useful tool for exploration of β_3_-AR-related pathophysiology.

β_3_-AR signaling has been highlighted to exert a neuroprotective function in multiple neurodegenerative models. In animals with 6-hydroxydopamine-induced lesions of noradrenergic neurons in the locus coeruleus, norepinephrine activation of β_3_-ARs rescues olfactory deficits, cognitive deficits, and depressive-like behavior (Sampaio et al., 2019). Additionally, β_3_-ARs are critical for motor learning in rotarod tasks and blockade of cerebellar synaptic plasticity in Purkinje cells by β_3_-ARs pharmacological blockade, further emphasizing the importance of the receptor in cerebellar function and motor coordination (Lippiello et al., 2020).

Increased further research has indicated that activation of β_3_-AR modulates GABAergic neurotransmission within the prefrontal cortex layer V/VI. Activation of Adrb3 promotes inhibitory transmission via presynaptic and postsynaptic mechanisms. Presynaptic is the depolarization of somatostatin-expressing interneurons through inhibition of Kir channels, resulting in heightened Ca^2+^ influx through T-type voltage-gated calcium channels. Postsynaptically, stimulation of β_3_-AR increases the efficacy of GABAA receptors through a Gs/cAMP/PKA-dependent signal transduction process, complementing its role in cortical network modulation (Luo et al., 2025).

Pharmacological modulation of β_3_-ARs has shown potential in neuropsychiatric illness. The brain-permeable β_3_-AR agonist, SR58611A, produces anxiolytic- and antidepressant-like effects. Therefore, the ability of SR58611A to potentiate the release of glutamate has been demonstrated, and the molecular mechanism by which this occurs, through the Ca^2+^/calmodulin/myosin light-chain kinase/myosin II cascade, has recently become clear (Wang et al., 2021).

Collectively, these studies highlight the diverse roles of β_3_-ARs across a range of metabolic, neurotransmission, and synaptic plasticity. Their involvement in neuroprotection, motor learning, and regulation of emotional response suggests their therapeutic potential as a target for neurodegenerative and psychiatric illnesses. Modulation of β_3_-ARs should remain a focus of research in future studies as a way of rescuing neuronal function and treating the root causes of disease progression.

### Alpha-adrenergic receptors

Alpha-adrenergic receptors (α-ARs) have a multidimensional role in synaptic function, AD, and neuroprotective and destructive roles. Stimulation of α_1_-AR maintains synaptic function, inhibits neuroinflammation, and has positive cognitive outcomes (Perez, 2023). The selective α_2_-AR agonists also confer neuroprotection by preventing amyloidogenic activity and stimulating neurogenesis. Elevated circulating agonistic α_1_-AR autoantibodies in certain AD patients cause continuous overstimulation, eventually resulting in neurotoxicity and vascular pathology (Hempel et al., 2016; Karczewski et al., 2018). Activation of α_2_-AR may also cause amyloid formation and pathological APP processing (Chen et al., 2014).

α_1_b-AR knockout mouse studies underscore its position in cognition, since deletion of the receptor prevents memory and discovery (Knauber and Muller, 2000). Blocking α_1_-AR, however, is also of therapeutic benefit. Doxazosin protects against Aβ toxicity through glycogen synthase kinase 3 beta activation inhibition and tau hyperphosphorylation, while prazosin prevents Aβ aggregation, inflammation, and memory impairment (Katsouri et al., 2013; Coelho et al., 2019). Terazosin was shown to elevate ATP levels and trigger autophagy, thereby reducing pathological protein aggregates and enhancing behavioral deficits (Chen et al., 2023). These findings indicate that selective inhibition of α_1_-AR can reduce AD pathology (Yu et al., 2022).

In addition to therapeutic value, activation of α_1_-AR is associated with vascular dysfunction, amyloid disease causation, and autoantibody-exacerbated behavior abnormalities (Pohlmann et al., 2014; Karczewski et al., 2018). Immunoadsorption of α_1_-AR autoantibodies preserves cognition in patients with AD, showing pathological participation during the disorder (Hempel et al., 2016; Karczewski et al., 2018). Upregulation of α_1_-AR is also associated with aggression in AD, suggesting further dysregulation-mediated pathogenesis of disease (Sharp et al., 2007).

α_2_-ARs also have a dual role in AD. Whereas α_2_b-AR deletion is associated with enhanced memory and reduced AD risk, activation of α_2_A-AR increases production of Aβ (Koutroumani et al., 2013; Chen et al., 2014). AD brains show a 50% reduction of α_2_-AR density in the prefrontal cortex, consistent with noradrenergic synapse loss (Kalaria and Andorn, 1991; Pascual et al., 1992).

Overall, α-AR modulation offers a compelling but complicated therapeutic potential in AD. Selective α_1_-AR blockade and α_2_-AR blockade could potentially be neuroprotective, but more investigation will be required to optimize therapeutic interventions based on these receptors.

### Advantages of β_2_-adrenergic receptors

Within β-adrenergic receptors, β_2_-ARs confer distinct synaptic and neuroprotective advantages. In contrast to β_1_-ARs, which mainly modulate responses to stress and cardiac function (Gupta et al., 2019; de Moura et al., 2023; Xu et al., 2024; Pyle, 2025), β_2_-ARs are highly abundant in hippocampal and cortical networks, where they boost LTP and block damage by Aβ oligomer to synapses (Li et al., 2013; Jin et al., 2023). In contrast, β_1_-AR activation diversely influences synaptic resilience and can exacerbate excitotoxicity during neurodegenerative conditions (Osborne et al., 1999; Chidlow et al., 2000).

β_2_-ARs are of more cognitive value than β_1_-ARs, which deplete working memory. Stimulation of β_2_-ARs strengthens working memory by modulating sustained neuronal discharge in the prefrontal cortex (Ramos et al., 2005, 2008). Unlike β_3_-ARs, which have a negligible CNS impact and occur mostly in adipose tissue, β_2_-ARs have direct synaptic plasticity effects aside from neuroprotection. Though α_2_-ARs improve working memory with moderate concentrations of norepinephrine, their action is suppressed under stress. β_2_-ARs, on the other hand, have cognitive advantages without stress-induced impairments and thus are the most promising β-adrenergic target for AD treatment.

For their function on immunomodulation, β_2_-ARs are more anti-inflammatory than β_1_-ARs by downregulating NF-κB signaling and polarizing microglia towards the anti-inflammatory phenotype. β_3_-ARs display peripheral anti-inflammatory effects without remarkable CNS expression or synaptic activities (Aboulata and Shatla, 2024; Nayak et al., 2024). Additionally, β_2_-ARs uniquely regulate epigenetic events by preventing HDAC2/3 inhibition, with synapse protection against gene silencing, an unmet β_1_- and β_3_-AR function. These interactive functions make β_2_-ARs a more deserving therapeutic drug in AD to demonstrate an advantage towards synaptic plasticity, neuroinflammation, and cognitive resilience.

**Figure 4:**
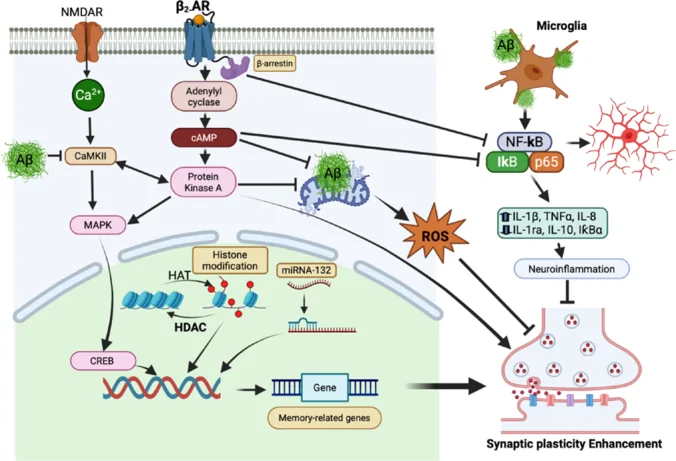
β_2_-AR signaling activation inhibits soluble Aβ oligomer-induced synaptic impairment. β_2_-AR activation activates the cAMP/PKA pathway, and it reverses Aβ-disrupted function of CaMKII and ERK signaling, leading to increased synaptic plasticity. In addition, elevated intracellular cAMP or β-arrestin signaling prevents Aβ-induced microglia-mediated neuroinflammation, the major driver of synaptic dysfunction. Moreover, cAMP/PAK activation prevents Aβ-dysfunctional mitochondrial function and induces synaptic plasticity by epigenetic control, i.e., histone modification and miRNA. Created with BioRender.com. β_2_-AR: β_2_-Adrenergic receptor; CaMKII: Calcium/calmodulin-dependent protein kinase II; cAMP: cyclic adenosine monophosphate; CREB: cAMP response element binding protein; HAT: histone acetyltransferase; HDAC: histone deacetylase; IL: interleukin; IL-1ra: interleukin-1 receptor antagonist; IκB: inhibitor of kappa B; MAPK: mitogen-activated protein kinase; NF-κB: nuclear factor kappa B; NMDAR: N-methyl-D-aspartate receptor; ROS: reactive oxygen species; TNFα: tumor necrosis factor alpha.

β_2_-AR seems to pop up as the top candidate when thinking about treating AD. Instead of being stuck with those peripheral effects like we see with β1-AR and α-adrenergic receptors, which, let us face it, rarely offer solid neuroprotection, activating β_2_-AR gets right to the heart of the problem. It addresses the complex issues in AD, including erratic synapse function, episodes of neuroinflammation, mitochondrial dysfunction, and unusual changes in gene regulation (**Figure 4** and **Table 1**). In most cases, these standout features hint that targeting β_2_-AR with agonists could be a promising path toward treatments that do not just ease the symptoms but might change the way the disease unfolds.

**Table 1:** Influence of β_2_-AR activity on the progression of AD and other neurodegenerative diseases

Mechanism/pathological target	β2-AR effects	Evidence/models	Therapeutic agonists	References
Synaptic plasticity (LTP/LTD)	Enhances LTP; suppresses LTD; promotes AMPA/NMDA receptor trafficking and CREB-BDNF expression	Aβ oligomer-treated slices; APP/PS1, 5×FAD mice; enriched environment exposure	Clenbuterol, formoterol, salmeterol	Li et al., 2013; Tanaka et al., 2020; Jin et al., 2022, 2023; Larsen et al., 2023; Hajiaghayi et al., 2024; Midorikawa et al., 2024; Prinkey et al., 2024
Aβ pathology	Reduces Aβ-mediated synaptic impairment; enhances microglial phagocytosis and clearance	Transgenic AD models; cell cultures	Clenbuterol, formoterol	Wang et al., 2010, 2011; Chai et al., 2016, 2017, 2022; Jin et al., 2023; Evans et al., 2024; Le et al., 2025
Tau pathology	Decreases tau hyperphosphorylation; promotes autophagy-mediated clearance	Tauopathy mouse models; hippocampal neurons	Clenbuterol	Deng et al., 2019; Lee et al., 2020; Lazzeri et al., 2021; Chai et al., 2022; Wu et al., 2024
Neuroinflammation	Polarizes microglia to anti-inflammatory phenotype (M2); reduces pro-inflammatory cytokines (TNF-α and IL-6)	AD mouse models; microglial cultures	β2-AR agonists	Heneka et al., 2010; Xu et al., 2018; Sharma et al., 2019; Damo et al., 2023; Jin et al., 2023; Evans et al., 2024
Epigenetic regulation	Inhibits HDAC2/3; increases histone acetylation; upregulates miRNA-132; promotes gene transcription	Cell cultures; rodent models	β2-AR agonists	Park et al., 2011; Mittal et al., 2017; Wei et al., 2020; Chai et al., 2022; Jin et al., 2023
Mitochondrial function	Promotes mitochondrial biogenesis (PGC-1α induction); restores fusion-fission balance; stabilizes Ca2+ flux	Neuronal cultures; rodent models	Formoterol, clenbuterol	Miura et al., 2007; Arif et al., 2019; Scholpa et al., 2019; Vekaria et al., 2020; Chang et al., 2024
Other neurodegenerative diseases (e.g., Parkinson’s disease)	Preliminary evidence suggests neuroprotection via synaptic and inflammatory mechanisms	Various preclinical neurodegeneration models	Under investigation	Bartus et al., 2016; Mittal et al., 2017; Giorgianni et al., 2020; Khidr et al., 2023; Chang et al., 2024; Inchiosa 2024; Hu et al., 2024

AD: Alzheimer’s disease; AMPA: α-amino-3-hydroxy-5-methyl-4-isoxazolepropionic acid; Aβ: amyloid-beta; β2-AR: β2-adrenergic receptor; BDNF: brain-derived neurotrophic factor; CREB: cyclic AMP response element-binding protein; IL: interleukin; LTD: long-term depression; LTP: long-term potentiation; NMDA: N-methyl-D-aspartate; PGC-1α: peroxisome proliferator-activated receptor-gamma coactivator-1α; TNF-α: tumor necrosis factor alpha.

## Future Directions and Challenges

Growing evidence justifying β_2_-AR activation as a therapeutic approach in AD reinforces the justification for targeted development. Although preclinical models validate their potential to enhance synaptic integrity, ameliorate neuroinflammation, and promote cognitive precursors of resilience, several key issues remain to be addressed before β_2_-AR agonists can be considered a clinical reality.

### Optimizing central nervous system targeting of β_2_-adrenergic receptor agonists

A key challenge in the clinical development of β_2_-AR agonists for neurological applications is achieving effective CNS penetration without inducing systemic side effects, particularly cardiovascular toxicity. Salmeterol and formoterol, the most clinically applied β_2_-AR agonists, are weakly permeable through the BBB and thus have poor CNS efficacy. Peripherally active drugs such as salbutamol show minimal BBB penetration and are hence of no usefulness in treating central β_2_-ARs. To reverse this, the future of drug development will target brain-selective β_2_-AR agonists with greater BBB permeability and less peripheral activity. There are some promising approaches: (1) CNS-specific agonist design from advances in medicinal chemistry may lead to molecules with greater selectivity for the brain. (2) Prodrug strategies, such as bio-oxidizable or light-activated (optopharmacological) systems, might enable targeted activation in the CNS with reduced peripheral exposure (Bohn et al., 2015; Axelrod et al., 2023; Sink et al., 2024). (3) Novel delivery platforms, including nanocarrier systems or intranasal delivery, may make brain-specific delivery viable (Adnet et al., 2020; Groo et al., 2025). These strategies collectively provide a means to more effective and safer CNS-targeted β_2_-AR therapies.

### Mechanistic elucidation and biomarker identification

An integrated knowledge of β_2_-AR signaling and cross-talk with synaptic plasticity, epigenetic regulation, neuroinflammation, mitochondrial dynamics, tau pathology, and Aβ homeostasis is necessary for the optimization of AD therapeutics. While β_2_-AR activation has been shown to affect synaptic function and modulate neuroinflammatory responses, the molecular mechanism is poorly understood. Data indicate β_2_-AR signaling can modulate synaptic genes via HDAC2/3-mediated epigenetics and drive an increase in a neuroprotective microglial phenotype. These results highlight the importance of mapping cross-talk among β_2_-AR pathways and crucial cellular processes. High-resolution multi-omics and functional assays will be critical to identify new targets and define the role of β_2_-AR in disease pathology. Concurrently, the discovery of robust biomarkers, i.e., miRNA signatures, epigenetic signatures, or imaging markers of synaptic integrity, will allow patient stratification and dynamic monitoring of therapeutic response. These mechanistic endpoints are critical to maximize β_2_-AR–based therapies and translate them effectively to the clinic.

### Timing and disease stage targeting

Since AD is a progressive condition, the therapeutic efficacy of β_2_-AR agonists should also be a function of the disease stage, with maximum effect potentially occurring in early or prodromal stages with synaptic dysfunction before widespread neurodegeneration sets in. Use in early stages may potentially salvage the synapses and decelerate the progression of the disease. Long-term observation of preclinical and human cohorts will be necessary to establish this optimal window of treatment and evaluate the possible efficacy of late-stage treatment. Moreover, evaluating whether chronic activation of β_2_-AR results in receptor desensitization or decreased efficacy with prolonged time will be fundamental to the design of long-term therapeutic regimens.

### Clinical translation and safety considerations

While preclinical findings are promising for the therapeutic use of β_2_-AR agonists in AD, numerous large-scale challenges need to be addressed initially before these drugs can be practically pushed into the clinic. One of these large challenges is tolerability among the elderly because AD patients are likely to have comorbidities such as cardiovascular disease and hypertension. Such conditions can increase the risk for side effects of β_2_-AR agonists, and thus thorough safety assessments must be carried out to avoid worsening peripheral pathologies. Clinical trial design must also be optimized to the maximum degree to identify both cognitive and functional benefits. Incorporation of biomarker-directed approaches, e.g., assessing decreases in neuroinflammation or changes in markers of synaptic plasticity, can augment sensitivity to treatment effects beyond traditional means of measuring cognition. Just as vital is the establishment of reliable biomarkers that effectively quantify central β_2_-AR activation and its neuroprotective effect in AD patients, whereby therapeutic response can be gauged and more tailored treatment methods can be instituted.

### Preventing receptor desensitization

Long-term stimulation of β_2_-AR has been implicated in receptor desensitization by mostly β-arrestin recruitment and subsequent internalization (Tokmakova et al., 2022). To preserve receptor sensitivity and widen the therapeutic window, various strategies have been proposed. These encompass dosing schedules providing time intervals between doses for receptor resensitization, co-administration of selectively β-arrestin-modulating, yet not desensitizing, stimulating useful signaling cascades, use of phosphodiesterase inhibitors to obtain maximal downstream signaling (Houslay and Baillie, 2005; Bruss et al., 2008), and biased agonist design for selective stimulation of neuroprotective cascades with minimal involvement in receptor downregulation. Together, these strategies seek to maintain long-term β_2_-AR efficacy and maximize therapeutic gain in chronic treatment.

### Combinatorial approaches

Since the etiology of AD is multifactorial, β_2_-AR agonists are optimally indicated in combination treatments. Their therapeutic efficacy would be most likely improved by a combination with compounds targeting complementary pathogenetic mechanisms, such as anti-amyloid or anti-tau treatment, anti-inflammatory drugs, or mitochondrial activators, and thus inducing synergistic neuroprotection. One of the most promising strategies is the synthesis of multi-target-directed ligands, or pleiotropic ligands, which combine multiple pharmacologically active moieties onto a single molecule. This strategy allows for the simultaneous modulation of β_2_-AR signaling and other important mechanisms of disease in the hope of increasing efficacy with fewer side effects typically experienced from polypharmacy. The rational development of such ligands to act on both β_2_-ARs and other AD-related mechanisms is a promising area for therapeutic intervention (Travers-Lesage et al., 2022).

### Broader applicability in neurodegeneration

The beneficial effects of β_2_-AR signaling are not limited to AD and can potentially be applied to other neurodegenerative diseases with similar synaptic dysfunction and inflammation, including Parkinson’s disease, Huntington’s disease, and frontotemporal dementia. Preclinical validation in these diseases could expedite wider therapeutic adoption.

The neuroprotective and immunomodulatory action of β_2_-AR signaling should also be explored in much more detail on a wide range of neurodegenerative diseases with common pathological features such as synaptic dysregulation, persistent neuroinflammation, and activation of glia. These conditions comprise Parkinson’s disease, as typified by progressive loss of dopaminergic neurons and synaptic damage (Alvarez-Luquin et al., 2025); Huntington’s disease, with resulting excitotoxicity and corticostriatal synaptic plasticity (Rana et al., 2024); and frontotemporal dementia, as typified by rapid synaptic breakdown and inflammatory responses (Bright et al., 2019; Clayton et al., 2024). Analysis of the function of β_2_-AR signaling within these models can reveal common mechanisms of neural resilience and immune control. Preclinical confirmation of β_2_-AR-targeted therapy in such conditions may not only further support therapeutic translation but also enable the creation of broad-spectrum neuroprotective approaches with potential applicability to various dementia and neurodegeneration.

## Conclusion and Perspective

With more supporting strands of evidence emerging, the β_2_-AR stands out as a promising therapeutic AD target. From facilitating synaptic plasticity and reducing neuroinflammation to epigenetically regulating these events and even acting at the mitochondria, β_2_-AR signaling has far-reaching neuroprotective properties. Activation of β_2_-AR stimulates long-term potentiation, rescues synaptic dysfunction induced by amyloid-beta and tau, and increases neurotrophic factor and anti-inflammatory cytokine in the bloodstream and CNS. Notably, β_2_-AR signaling is employed in resilience against cognitive deficits following exposure to enriched environments, suggesting a biological role for the receptor. However, clinical translation is complicated by key translational obstacles: penetrability through the blood–brain barrier, receptor desensitization, and off-target effects. Optimized pharmacologic delivery and CNS-selective β_2_-AR agonists are major advancements. Moreover, combinatorial therapies with β_2_-AR targets and those targeting amyloid, tau, and mitochondrial dysfunction may work synergistically. Armed with an enhanced understanding of the pathways mediated by β_2_-AR, particularly the epigenetic and immunomodulatory functions, it is possible to fabricate new treatment avenues to more effectively engage with AD pathophysiology earlier and in a more malleable way. Ultimately, manipulation of β_2_-AR signaling can not only halt AD pathology but also reverse it with great potential for other neurodegenerative conditions.

## Data Availability

*Not applicable.*
